# Bone Marrow Derived CD34 + cells and Leukocytes in 729 Children and Adults with Non‐malignant Diseases

**DOI:** 10.1007/s12015-021-10173-3

**Published:** 2021-05-02

**Authors:** Christof Pabinger, Brenda Laky, Philipp R. Heuberer, Georg S. Kobinia

**Affiliations:** 1 Institute for Regenerative Medicine (IRM), Graz, Austria; 2 Medical University of Innsbruck, Innsbruck, Austria; 3 Austrian Society of Regenerative Medicine, Wollzeile 3/ Top 2.1, 1010 Vienna, Austria; 4 Austrian Research Group for Regenerative and Orthopedic Medicine (AURROM), Vienna, Austria; 5 OrthoCare and HealthPi Medical Center, Vienna, Austria

In the past decade, bone marrow (BM)-derived cell-based therapies have been increasingly performed in various fields of regenerative medicine. Yet, little is known about standard values of these BM cells. Furthermore, CD34 + cell counts, markers for multipotent hematopoietic stem cells, are still not routinely reported in therapeutic studies with autologous BM. Thus, crucial knowledge of what we are doing in regenerative medicine is at least in part lacking. The reason for this discrepancy may be that hematooncologist, who have the best knowledge in BM physiology, rarely engage in regenerative medicine, while on the other side, specialists (mainly surgeons) engaged in regenerative medicine lack specialization in hematology.

Autologous BM-derived cell-based therapies in regenerative medicine are on the rise. While it is generally known that BMA contains a mix of nucleated cells and other biologics such as growth factors and exosomes, comprehensive information regarding normal ranges of leucocytes and/or CD34 + cell counts in BMA in a large series of patients of all age groups was missing.

Stem cells are mononuclear cells and therefore, a fraction of the BM-derived leukocytes. Surprisingly, to our knowledge no prior study described the positive and strong correlation between both parameters, which might be of use in future clinical practice. Since stem cells can only be identified using specific CD antigen sets (CD34, CD90, CD45, CD107,…), which is costly and laborious, this correlation can be utilized, to predict the amount of stem cells based on the number of BM-derived leukocytes alone, which is much easier. Using the newly described correlation, a much cheaper possibility exists, to assess, if a specific patient has a high or low stem cell number. It has to be assessed in the future, if patients with a higher leukocyte- and stem cell- count will have better outcomes and might therefore be better suitable for stem cell operations.

However, stem cell counts are important, since it is generally assumed that better clinical results can be achieved with a higher donor-site stem cell count [1], especially for hematopoietic stem cell transplantation where the volume of graft required is commonly based on either the number of CD34 + cells or the number of total nucleated BM cells [2]. In addition, BM stem cell counts may serve as a maker for severity of disease.

To be able to compare various point-of-care autologous stem cell procedures, and thus, to optimize techniques, similar to studies reporting cell counts after applying expansion techniques, the major aim of this project was the attempt to define reference values for cell counts in BM, especially in children.

With the present study, we are reporting counts of CD34 + cells and leukocytes in BMA in a large number of children (n = 445) and adult (n = 284) patients who underwent autologous point-of-care stem cell transplantation for various non-malignant diseases. Cell counts in BMA and centrifuged BMA-concentrate were also analyzed regarding influences of gender and diagnose related groups (Table S1).

The median percentage of CD34 + cells in BMA leukocyte counts was 1.1 %; which was significantly higher in children aged between 2 and 18 years (1.38 %; IQR 1.03–1.77) than in adults (0.62 %; IQR 0.38–0.88; p < 0.001).

Very strong correlations were detected between age (years) and CD34 + cells (count/µl/kg) in BMA (Fig. 1; rho = -0.827, p < 0.001). All correlations between age and cell counts are presented in Table S2.

**Fig. 1:**
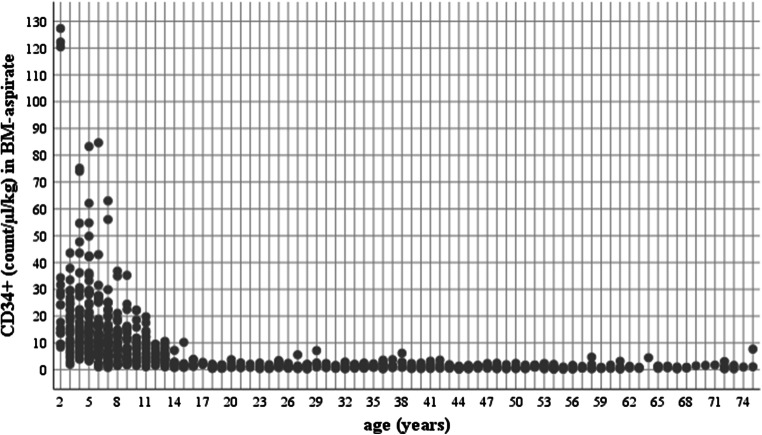
CD34 + cells and age. Scatter plot showing the relationship between CD34 + cells (count/µl/kg body weight) and age (years) in bone marrow (BM) aspirate

Children (9.8, IQR 5.1–16.6) had significantly more CD34 + cells/µl/kg than adults (1.0, IQR 0.7–1.6; p < 0.001) in BMA. Furthermore, comparison regarding CD34 + cells (count/µl) in BMA showed significant differences between age groups (p < 0.001), while no significant differences were detected between adults’ age groups (19-39years vs. 40-59years: p = 0.326; 19-39years vs. 60-79years: p = 0.874; 40-59years vs. 60-79years: p = 0.999).

Comparisons between gender and diagnose related groups regarding BMA and BMA concentrate showed significant differences between diagnose related groups, but not between females and males (Table S3).

Results in the assessment of the association between age and stem cell counts may be influenced by the underlying disease of the patients. Hence, the results of such studies are controversial [3, 4]. Obviously, large samples in healthy people will hardly ever be collected.

According to our data stem cell counts do not deteriorate in adults (18–75 years); this supports the work by Povsic et al.[5] who reported that ageing is not associated with BM-resident progenitor cell depletion (18–85 years).

Yet, we are unaware of any study reporting data of cell counts in children (2-18years). Despite the fact that cell counts of our large group of children had several different diagnoses, leucocytes and CD34 + cell counts were significantly lower in adults. It remains speculative, if the higher cell count in children is necessary for growth and differentiation up to the age of puberty. However, since cell counts remained stable in adults (18-75years), increasing age might not necessarily be a contraindication for autologous stem cell therapy.

It is also not clear yet, if heterogeneous clinical results are linked to heterogeneous BM-derived stem cell counts. One could assume, that a higher yield of stem cells might result in a superior clinical outcome. Anyway, the possible predictive value of stem cell counts needs further investigations, especially linking donor-site cell counts to clinical outcome and thus, to sort out patients with unfavorable cell counts to avoid unnecessary interventions.

Despite the large sample size reporting cell counts, there are some limitations: Cell counts are probably not comparable to individuals with other diagnoses than those reported. Severity of illness might also contribute to cell count variations and hence, influence data. Cell counts might also be influenced by BMA volume, which we did not measure in this study. Further studies of different age groups are needed to evaluate typical BM-derived cell counts of individuals with other diagnoses and to report cell count related outcome. However, this large study provides baseline data showing that BM-derived CD34 + cells were significantly higher in younger patients, while similar cell counts were detected within adults for further evaluations regarding autologous BM-derived cell therapies.

## Supplementary Information

ESM 1 (DOCX 308 KB)

## Data Availability

Datasets supporting the conclusions of this article are included within the article and its additional file.
