# Supplementary material for: Bone Marrow Derived CD34 + cells and Leukocytes in 729 Children and Adults with Non‐malignant Diseases
Source: Stem Cell Rev Rep. 2021 May 2;17(4):1508–10. doi: 10.1007/s12015-021-10173-3 (PMC8316224; doi:10.1007/s12015-021-10173-3)
Supplement: Supplementary file 1 — (DOCX 308 KB) [file 12015_2021_10173_MOESM1_ESM.docx]

**SUPPLEMENT**

**Bone marrow derived CD 34+ cells and leukocytes in 729 children and adults with non-malignant diseases**

**SUPPLEMENTARY ABSTRACT**

**Background:** To our knowledge, there are no large studies reporting bone marrow (BM)-derived cell counts of children and adults with non-malignant diseases. Thus, the primary objective was to evaluate BM-derived CD34+ and leukocyte cell counts in 729 patients of different age groups who underwent point-of-care autologous cell-based therapy for non-malignant diseases.

**Methods:** For this study, a retrospective data analysis of laboratory parameters including BM-aspirated and post-centrifuge concentrated CD34+ cells and leukocytes was performed. Associations and differences of cell counts between age groups, gender, and diagnose related group were evaluated.

**Results:** Included were data of 187 female and 542 male patients aged between 2 and 75 years, who underwent autologous cell-based therapy. Children (median 9.8, range [5.1-16.6]) had significantly more CD34+ cells/µl/kg than adults (median 1.0, range [0.7-1.6]; p<0.001). The median percentage of CD34+ cells of leukocytes was 1.10% in BM-aspirate (BMA) and 0.96% in post-centrifuge BMA-concentrate (BMAC). Significant moderate positive correlations were observed between CD34+ cells (count/µl) and leukocytes (count/µl) in BMA and BMAC. Significant strong negative correlations were detected between age (years) and CD34+ cells (count/µl/kg) in BMA and BMAC. No significant differences regarding CD34+ cells (count/µl) in BMA were detected between adults, while significant differences regarding cell counts were detected between diagnose related groups, but not between females and males.

**Conclusions:** This study demonstrated BM-derived CD34+ and leukocyte cell counts in BMA and BMAC of 729 patients with various non-malignant diseases. While BM-derived CD34+ cells were significantly higher in younger patients, similar cell counts were detected within adults.

**Keywords:** CD34+ cells, leukocytes, age, children, adults, bone marrow, autologous, stem cell

**SUPPLEMENTARY MATERIAL AND METHODS**

For this retrospective study, all laboratory data of patients who underwent stem cell transplantation for non-malignant diseases up to 2019 were evaluated. The respective diagnoses of patients treated at the institute for regenerative medicine were spinal cord injury, amyotrophic lateral sclerosis, autism, cerebral palsy, neurodegenerative diseases (e.g. multiple sclerosis, Parkinson, ataxia), muscle dystrophy, and neurological diseases (e.g. traumatic brain injury, stroke). Included were female and male patients aged between 2 and 75 years with available BM-derived CD34^+^ cells (count/µl and count/µl/kg body weight) and leukocytes (count/µl and cells/µl/kg body weight) of the BMA and post-centrifuged BMAC. Percentages of CD34^+^ cells of leukocytes and concentrations of BMAC of BMA were calculated. Data from procedures with missing or invalid cell counts were excluded. The study was approved by the ethics committee of the Medical University of Graz (31-152 ex 18/19, Ethikkommission der Medizinischen Universität Graz, Auenbruggerplatz 2, 1.OG, 8036 Graz, Austria / EU). All methods **were carried out in accordance with relevant guidelines and regulations**.

All procedures were performed as point-of-care method in a laminar air flow operating room. Aspiration of BM was performed by the same experienced surgeon (G.S.K.) from the posterior and anterior iliac crest. Stem cells were harvested with a Yamshidi Needle (15ga x 2.688in MAX Bone Marrow Aspiration Needle, ARGON Medical devices, Athens, USA) under sedoanalgesia. BMA was retrieved using 10ml syringes and changing direction repeatedly as published by Oliver et al.[1]. Following, BMA was processed in the operating room according to the SmartRedux protocol (Biosafe, Eysins, Switzerland) using a fully automated cell separator system (Sepax^®^ S-100; Cytiva Europe GmbH, Freiburg, Germany).

One ml of the total BMA sample was immediately transferred to the same laboratory and analyzed with fluorescence activated cell sorter (FACS) using a stem cell kit from Beckman Coulter and the ISHAGE protocol (<https://www.bc-cytometry.com/PDF/DataSheet/IM3630.pdf>).

**Statistical analysis**

Demographic details were presented using descriptive statistics. Data distribution was assessed by visual inspection of histograms and the Kolmogorov-Smirnov test. Qualitative data were expressed by numbers and percentages and quantitative data as means with standard deviation or median with range. CD34+ concentrations (in times) after centrifugation were calculated as CD34+ cells (count/µl) in BMAC divided by CD34+ cells (count/µl) in BMAC. For continuous and normal distributed data, independent t-tests were applied and Mann-Whitney U or Kruskal-Wallis tests were used for non-parametric data to determine differences between two or more groups, respectively. Bonferroni adjustments served for multiple testing. Spearman's rank correlation coefficients (rho) were used to assess the correlation between parameters. Statistical significance level was set at *P* < .05 (2-sided). Statistical analyses were performed using SPSS Statistics 25 (IBM Corporation, Armonk, NY).

**SUPPLEMENTARY RESULTS**

A total of 729 laboratory datasets were evaluated. There were significantly more children (n=445, 61.0%) than adults (n=284, 39.0%; p<0.001) and males than females (p<0.001) treated with autologous stem cell transplantation. Frequencies of CD34+ cells (count/µl) and leukocytes (count/µl) in BMA are presented in Supplement file Figure S1 and S2. The median percentage of CD34+ cells of the leukocytes in BMA as well as in BMAC was 1.1%.


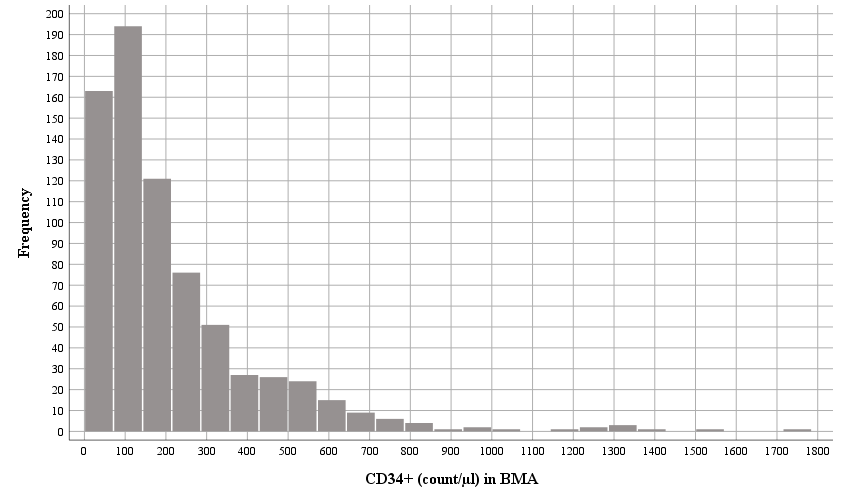


**Figure S1** Frequencies of CD34+ cells (count/µl) in bone marrow aspirate (BMA).


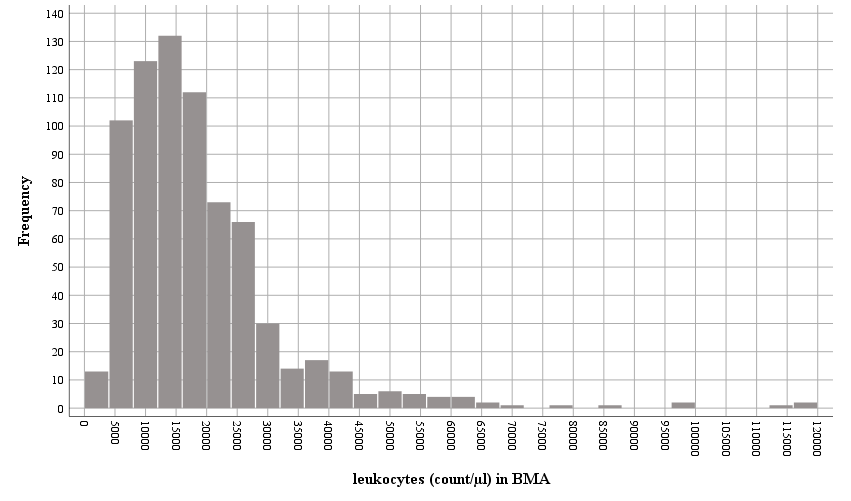


**Figure S2** Frequencies of leukocytes (count/µl) in bone marrow aspirate (BMA).

All demographic data are presented in Table S1.

**Table S1** Demographic data

| **Characteristics** | **TOTAL**  (n=729) |
| --- | --- |
| Age (years) at time of procedure  Mean ± SD  Median (range) | 21.1 ± 19.6  11.0 (2-75) |
| Age groups (n, %)  Children  Preschool (2-5 years)  School (6-12 years)  Adolescent (13-18 years)  Adults  Young (19-39 years)  Middle-aged (40-59 years)  Elderly (≥60 years) | 169 (23.2)  231 (31.7)  45 (6.2)  124 (17.0)  120 (16.5)  40 (5.5) |
| Gender (n, %)  Female  Male | 187 (25.7)  542 (74.3) |
| Diagnose related group (n, %)  spinal cord injury  amyotrophic lateral sclerosis  autism  cerebral palsy  neurodegenerative diseases  muscle dystrophy  neurological diseases | 151 (20.7)  97 (13.3)  263 (36.1)  149 (20.4)  37 (5.1)  19 (2.6)  13 (1.8) |
| Bone marrow aspirate (median, IQR)  Leukocytes (count/µl)  Leukocytes (count/µl/kg body weight)  CD34^+^ cells (count/µl)  CD34^+^ cells (count/µl/kg body weight)  Percentage of CD34^+^ cells of leukocytes (%) | 15800 (10200, 23300)  416 (192.7, 879.5)  146 (75, 274)  4.47 (1.22, 12.90)  1.10 (0.66, 1.55) |
| Post centrifuge concentrate (median, IQR)  Leukocytes (count/µl)  Leukocytes (count/µl/kg body weight)  CD34^+^ cells (count/µl)  CD34^+^ cells (count/µl/kg body weight)  Percentage of CD34^+^ cells of leukocytes (%) | 85000 (46600, 137350)  2174 (1124.3, 4024.3)  800 (374.5, 1392.5)  18.46 (7.69, 43.48)  0.96 (0.58, 1.47) |

Abbreviation: IQR, interquartile range.

CD34+ cells (count/µl) and leukocytes (count/µl) in BMA (Figure S3) and in BMAC showed moderate positive correlations (rho= 0.687; p<0.001 and rho= 0.663; p<0.001, respectively).

In BMAC the median percentage of CD34+ cells of the leukocytes was also significantly higher in children (1.28%; IQR 0.90, 1.74) than in adults (0.60%; IQR 0.41, 0.88; p<0.001).

**
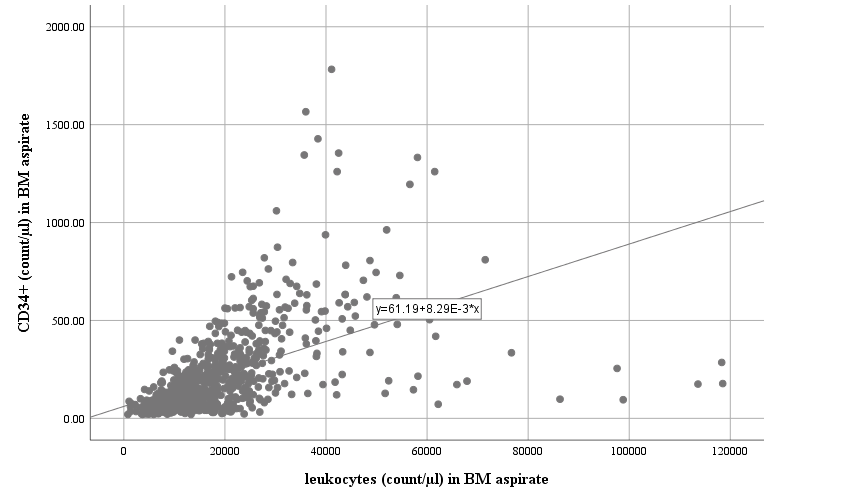
**

**FIGURE S3** CD34+ cells and leukocytes. Scatter plot showing positive linear relationship between CD34+ cells (count/µl) and leukocytes (count/µl) in bone marrow (BM) aspirate.

Strong negative correlations were detected between age (years) and CD34+ cells (count/µl/kg) in BMAC (rho = -0.712, p<0.001). The negative relationship between age (years) and CD34+ cells (count/µl) in BMA (rho= -0.601; p<0.001) was moderate and weak in BMAC (rho= -0.285; p<0.001). All correlations between age and cell counts are presented in Supplement file Table S2.

A comparison of CD34+ cells (count/µl/kg) in BMA between age-gender stratified groups showed significant differences between girls (9.3, range 0.8-42.9) and women (1.3, range 0.3-7.1; p<0.001), boys (10.0, range 0.5-127.3) and men (1.0, range 0.2-7.7; p<0.001), as well as between women and men (p=0.012), but not between girls and boys (p=0.513).

**Supplement Table S2** Correlations between age (years) and cell counts.

| Spearman's rho | | | | **Age**  **(years)** | **Bone marrow aspirate** | | | | | **Concentrate** | | | | | **BMA of**  **concentrate** |
| --- | --- | --- | --- | --- | --- | --- | --- | --- | --- | --- | --- | --- | --- | --- | --- |
|  |  |  |  |  | **CD34^+^** | | **Leukocyte** | | **CD34^+^ of**  **leukocytes** | **CD34^+^** | | **Leukocyte** | | **CD34^+^ of**  **leukocytes** |  |
|  |  |  |  |  | **(cells/µl)** | **(cells/µl/kg)** | **(cells/µl)** | **(cells/µl/kg)** | **(%)** | **(cells/µl)** | **(cells/µl/kg)** | **(cells/µl)** | **(cells/µl/kg)** | **(%)** |  |
| **Age (years)** | | | CC | 1.000 | -0.601^**^ | -0.827^**^ | -0.249^**^ | -0.762^**^ | -0.591^**^ | -0.285^**^ | -0.712^**^ | 0.144^**^ | -0.482^**^ | -0.570^**^ | 0.300^**^ |
|  |  |  | p-value | . | <0.001 | <0.001 | <0.001 | <0.001 | <0.001 | <0.001 | <0.001 | <0.001 | <0.001 | <0.001 | <0.001 |
|  |  |  | N | 729 | 729 | 729 | 729 | 729 | 729 | 729 | 729 | 729 | 729 | 729 | 729 |
| **Bone marrow aspirate** | **CD34^+^** | (cells/µl) | CC | -0.601^**^ | 1.000 | 0.903^**^ | 0.687^**^ | 0.777^**^ | 0.709^**^ | 0.520^**^ | 0.680^**^ | 0.127^**^ | 0.474^**^ | 0.552^**^ | -0.453^**^ |
|  |  |  | p-value | <0.001 | . | <0.001 | <0.001 | <0.001 | <0.001 | <0.001 | <0.001 | 0.001 | <0.001 | <0.001 | <0.001 |
|  |  |  | N | 729 | 729 | 729 | 729 | 729 | 729 | 729 | 729 | 729 | 729 | 729 | 729 |
|  |  | (cells/µl/kg) | CC | -0.827^**^ | 0.903^**^ | 1.000 | 0.528^**^ | 0.896^**^ | 0.738^**^ | 0.429^**^ | 0.786^**^ | -0.021 | 0.555^**^ | 0.622^**^ | -0.452^**^ |
|  |  |  | p-value | <0.001 | <0.001 | . | <0.001 | <0.001 | <0.001 | <0.001 | <0.001 | 0.571 | <0.001 | <0.001 | <0.001 |
|  |  |  | N | 729 | 729 | 729 | 729 | 729 | 729 | 729 | 729 | 729 | 729 | 729 | 729 |
|  | **Leukocyte** | (cells/µl) | CC | -0.249^**^ | 0.687^**^ | 0.528^**^ | 1.000 | 0.714^**^ | 0.043 | 0.307^**^ | 0.329^**^ | 0.288^**^ | 0.362^**^ | 0.090^*^ | -0.386^**^ |
|  |  |  | p-value | <0.001 | <0.001 | <0.001 | . | <0.001 | 0.242 | <0.001 | <0.001 | <0.001 | <0.001 | 0.015 | <0.001 |
|  |  |  | N | 729 | 729 | 729 | 729 | 729 | 729 | 729 | 729 | 729 | 729 | 729 | 729 |
|  |  | (cells/µl/kg) | CC | -0.762^**^ | 0.777^**^ | 0.896^**^ | 0.714^**^ | 1.000 | 0.406^**^ | 0.306^**^ | 0.683^**^ | 0.020 | 0.568^**^ | 0.424^**^ | -0.456^**^ |
|  |  |  | p-value | <0.001 | <0.001 | <0.001 | <0.001 | . | <0.001 | <0.001 | <0.001 | 0.590 | <0.001 | <0.001 | <0.001 |
|  |  |  | N | 729 | 729 | 729 | 729 | 729 | 729 | 729 | 729 | 729 | 729 | 729 | 729 |
|  | **CD34^+^**  **of**  **leukocytes** | (%) | CC | -0.591^**^ | 0.709^**^ | 0.738^**^ | 0.043 | 0.406^**^ | 1.000 | 0.421^**^ | 0.620^**^ | -0.102^**^ | 0.310^**^ | 0.698^**^ | -0.273^**^ |
|  |  |  | p-value | <0.001 | <0.001 | <0.001 | 0.242 | <0.001 | . | <0.001 | <0.001 | 0.006 | <0.001 | <0.001 | <0.001 |
|  |  |  | N | 729 | 729 | 729 | 729 | 729 | 729 | 729 | 729 | 729 | 729 | 729 | 729 |
| **Concentrate** | **CD34^+^** | (cells/µl) | CC | -0.285^**^ | 0.520^**^ | 0.429^**^ | 0.307^**^ | 0.306^**^ | 0.421^**^ | 1.000 | 0.816^**^ | 0.663^**^ | 0.710^**^ | 0.466^**^ | 0.482^**^ |
|  |  |  | p-value | <0.001 | <0.001 | <0.001 | <0.001 | <0.001 | <0.001 | . | <0.001 | <0.001 | <0.001 | <0.001 | <0.001 |
|  |  |  | N | 729 | 729 | 729 | 729 | 729 | 729 | 729 | 729 | 729 | 729 | 729 | 729 |
|  |  | (cells/µl/kg) | CC | -0.712^**^ | 0.680^**^ | 0.786^**^ | 0.329^**^ | 0.683^**^ | 0.620^**^ | 0.816^**^ | 1.000 | 0.354^**^ | 0.809^**^ | 0.631^**^ | 0.159^**^ |
|  |  |  | p-value | <0.001 | <0.001 | <0.001 | <0.001 | <0.001 | <0.001 | <0.001 | . | <0.001 | <0.001 | <0.001 | <0.001 |
|  |  |  | N | 729 | 729 | 729 | 729 | 729 | 729 | 729 | 729 | 729 | 729 | 729 | 729 |
|  | **Leukocyte** | (cells/µl) | CC | 0.144^**^ | 0.127^**^ | -0.021 | 0.288^**^ | 0.020 | -0.102^**^ | 0.663^**^ | 0.354^**^ | 1.000 | 0.702^**^ | -0.284^**^ | 0.548^**^ |
|  |  |  | p-value | <0.001 | 0.001 | 0.571 | <0.001 | 0.590 | 0.006 | <0.001 | <0.001 | . | <0.001 | <0.001 | <0.001 |
|  |  |  | N | 729 | 729 | 729 | 729 | 729 | 729 | 729 | 729 | 729 | 729 | 729 | 729 |
|  |  | (cells/µl/kg) | CC | -0.482^**^ | 0.474^**^ | 0.555^**^ | 0.362^**^ | 0.568^**^ | 0.310^**^ | 0.710^**^ | 0.809^**^ | 0.702^**^ | 1.000 | 0.110^**^ | 0.258^**^ |
|  |  |  | p-value | <0.001 | <0.001 | <0.001 | <0.001 | <0.001 | <0.001 | <0.001 | <0.001 | <0.001 | . | 0.003 | <0.001 |
|  |  |  | N | 729 | 729 | 729 | 729 | 729 | 729 | 729 | 729 | 729 | 729 | 729 | 729 |
|  | **CD34^+^**  **of**  **leukocytes** | (%) | CC | -0.570^**^ | 0.552^**^ | 0.622^**^ | 0.090^*^ | 0.424^**^ | 0.698^**^ | 0.466^**^ | 0.631^**^ | -0.284^**^ | 0.110^**^ | 1.000 | -0.073^*^ |
|  |  |  | p-value | <0.001 | <0.001 | <0.001 | 0.015 | <0.001 | <0.001 | <0.001 | <0.001 | <0.001 | 0.003 | . | 0.050 |
|  |  |  | N | 729 | 729 | 729 | 729 | 729 | 729 | 729 | 729 | 729 | 729 | 729 | 729 |
| **BMA of**  **concentrate** | | | CC | 0.300^**^ | -0.453^**^ | -0.452^**^ | -0.386^**^ | -0.456^**^ | -0.273^**^ | 0.482^**^ | 0.159^**^ | 0.548^**^ | 0.258^**^ | -0.073^*^ | 1.000 |
|  |  |  | p-value | <0.001 | <0.001 | <0.001 | <0.001 | <0.001 | <0.001 | <0.001 | <0.001 | <0.001 | <0.001 | 0.050 | . |
|  |  |  | N | 729 | 729 | 729 | 729 | 729 | 729 | 729 | 729 | 729 | 729 | 729 | 729 |

Abbreviations: BMA, bone marrow aspirate; CC, correlation coefficient;

**. Correlation is significant at the 0.01 level (2-tailed); *. Correlation is significant at the 0.05 level (2-tailed).

**Supplement Table S2.** Comparisons of cell counts between age groups, gender, and diagnose related groups

|  | **Bone marrow aspirate** | | | | | **Post-centrifuge concentrate** | | | | |  |
| --- | --- | --- | --- | --- | --- | --- | --- | --- | --- | --- | --- |
|  | **CD34^+^** | | **Leukocytes** | | **CD34^+^**  **of**  **leukocytes** | **CD34^+^** | | **Leukocytes** | | **CD34^+^**  **of**  **leukocytes** | **Concentration**  **of**  **BMA** |
|  | (cells/µl) | (cells/µl/kg) | (cells/µl) | (cells/µl/kg) | (%) | (cells/µl) | (cells/µl/kg) | (cells/µl) | (cells/µl/kg) | (%) |  |
| **Age groups** |  |  |  |  |  |  |  |  |  |  |  |
| Children  (n=445) | 226  [140, 382] | 9.8  [5.1, 16.6] | 17900  [1250, 25350] | 731.0  [421.0, 1121.1] | 1.34  [1.03, 1.77] | 940  [497.5, 1526.3] | 35.6  [18.8, 73.9] | 80300  [45550, 124550] | 3031.4  [1692.7, 4975.5] | 1.30  [0.90, 1.74] | 3.8  [2.2, 6.8] |
| Adults  (n=284) | 75  [47, 112] | 1.0  [0.7, 1.6] | 12800  [8275, 18500] | 176.0  [110.0, 271.8] | 0.62  [0.38, 0.88] | 520  [269, 1065] | 7.6  [3.9, 14.6] | 95700  [47400, 168950] | 1280.4  [702.2, 2345.9] | 0.60  [0.41, 0.88] | 6.9  [3.4, 13.8] |
| *P-value**  (children vs. adults) | <0.001 | <0.001 | <0.001 | <0.001 | <0.001 | <0.001 | <0.001 | <0.001 | <0.001 | <0.001 | <0.001 |
| **Gender** |  |  |  |  |  |  |  |  |  |  |  |
| Female  (n=187) | 144  [76, 255] | 3.7  [1.4, 11.8] | 15800  [9800, 23200] | 435.3  [244.2, 914.3] | 0.97  [0.63, 1.41] | 700  [340, 1313] | 19.4  [7.5, 45.9] | 82000  [41100, 134700] | 2200.0  [1139.1, 4464.3] | 0.96  [0.57, 1.45] | 4.6  [2.5, 8.8] |
| Male  (n=542) | 147  [75, 292] | 4.3  [1.1, 11.8] | 15750  [10375, 23350] | 410.3  [177.1, 879.2] | 1.08  [0.65, 1.57] | 821  [379, 1403] | 20.0  [8.4, 43.2] | 86550  [47375, 139150] | 2146.3  [1118.9, 3929.9] | 0.99  [0.62, 1.56] | 4.8  [2.5, 8.9] |
| *P-value**  (female vs. male) | 0.386 | 0.680 | 0.779 | 0.185 | 0.229 | 0.114 | 0.953 | 0.446 | 0.416 | 0.318 | 0.730 |
| **DRG** |  |  |  |  |  |  |  |  |  |  |  |
| spinal cord injury  (n=151) | 91  [58, 144] | 1.2  [0.8, 2.1] | 13600  [9800, 18500] | 201.3  [132.9, 306.9] | 0.69  [0.45, 0.93] | 680  [325, 1300] | 10.3  [4.7, 19.5] | 108800  [55300, 177500] | 1660.0  [818.8, 2900.0] | 0.68  [0.47, 0.95] | 6.9  [3.5, 14.3] |
| amyotrophic lateral sclerosis  (n=97) | 73  [40, 118] | 0.9  [0.6, 1.6] | 12100  [7800, 18450] | 172.2  [106.4, 301.6] | 0.63  [0.33, 0.83] | 470  [281, 839] | 6.7  [3.8, 12.5] | 97400  [43150, 162550] | 1285.4  [704.1, 2290.7] | 0.53  [0.32, 0.81] | 6.6  [2.9, 14.6] |
| autism  (n=263) | 270  [174, 440] | 10.2  [5.0, 16.7] | 19600  [14300, 26700] | 722.9  [416.7, 1062.5] | 1.38  [1.03, 1.78] | 940  [520, 940] | 32.3  [17.0, 58.0] | 74500  [42500, 116800] | 2336.1  [1453.8, 4150.0] | 1.32  [0.97, 1.85] | 3.4  [2.1, 5.7] |
| cerebral palsy  (n=149) | 183  [107, 273] | 10.2  [5.5, 16.4] | 14100  [9800, 22400] | 818.2  [439.4, 1201.1] | 1.31  [0.98, 1.79] | 950  [412, 1524] | 49.4  [21.7, 93.1] | 90900  [52300, 128000] | 4215.4  [2208.5, 7336.5] | 1.22  [0.76, 1.67] | 5.0  [2.1, 8.6] |
| neurodegenerative diseases  (n=37) | 70  [41, 108] | 1.0  [0.5, 1.8] | 13300  [7450, 20800] | 193.8  [96.0, 298.9] | 0.60  [0.35, 0.98] | 455  [229, 1051] | 7.1  [2.5, 15.6] | 79700  [41150, 174500] | 1004.0  [557.8, 2419.4] | 0.68  [0.26, 0.91] | 6.0  [3.9, 10.6] |
| muscle dystrophy  (n=19) | 101  [54, 238] | 3.2  [1.8, 6.2] | 14300  [10300, 25400] | 390.8  [300.0, 851.9] | 0.77  [0.61, 1.36] | 790  [385, 1395] | 25.5  [7.4, 39.9] | 88500  [64300, 139700] | 2957.1  [1268.0, 4296.3] | 0.95  [0.64, 1.38] | 6.5  [2.6, 10.8] |
| neurological diseases  (n=13) | 58  [45, 116] | 0.96  [0.77, 2.47] | 9700  [6750, 12700] | 188.7  [100.2, 270.2] | 0.90  [0.64, 1.05] | 300  [147, 670] | 6.4  [2.8, 13.5] | 32800  [26150, 63750] | 618.9  [395.5, 1522.8] | 0.79  [0.61, 1.18] | 4.4  [2.5, 9.5] |
| *P-value***  (within DRG groups) | <0.001 | <0.001 | <0.001 | <0.001 | <0.001 | <0.001 | <0.001 | <0.001 | <0.001 | <0.001 | <0.001 |

Abbreviation: DRG, diagnose related groups.

Data are shown as median [interquartile range]. *Mann-Whitney U test; **Kruskal Wallis test

Percentages of CD34+ cells of the leukocytes in BMA of children and adults according to their non-malignant diseases are presented in Figure S3.

**
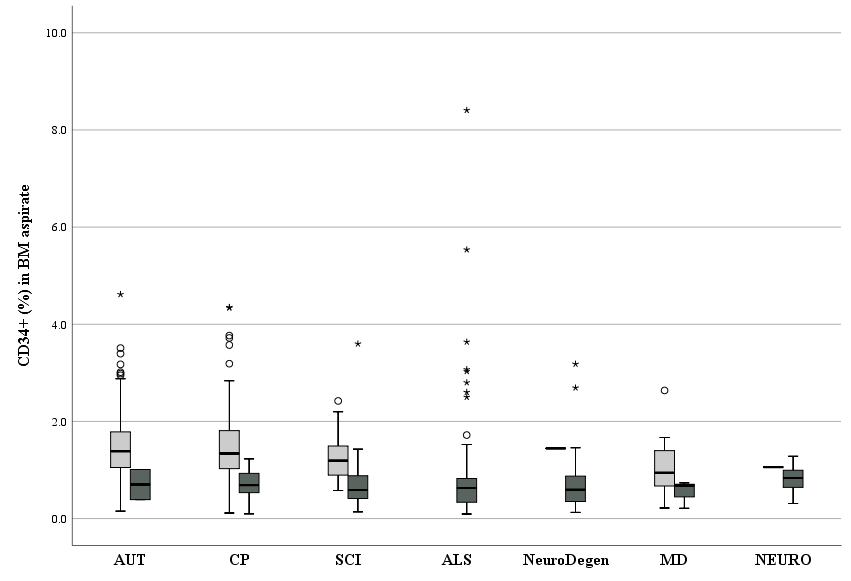
**

**FIGURE S3** CD34+ cells of patients with non-malignant diseases. Percentage of CD34+ cells of the leukocytes (count/µl) in bone marrow (BM) aspirate according to the non-malignant diseases including autism (AUT), cerebral palsy (CP), spinal cord injury (SCI), amyotrophic lateral sclerosis (ALS), neurodegenerative diseases (NeuroDegen), muscle dystrophy (MD), and neurological diseases (NEURO) of children (2-18 years, light grey) and adults (dark grey).

Density gradient separation using a centrifuge-based system showed that the lower the CD34+ cells (count/µl) in BMA, the higher the concentration (times) of CD34+ cells (count/µl) in BMAC after centrifugation (Figure 4) and thus, the system was able to increase the concentration of nucleated cells by a median of 4.59 (IQR 2.53, 8.85) times. Significant lower median concentration increase regarding CD34+ cells was detected in children (3.82, IQR 2.18, 6.81) than in adults (6.92, IQR 3.39, 13.8; p<0.001).

**
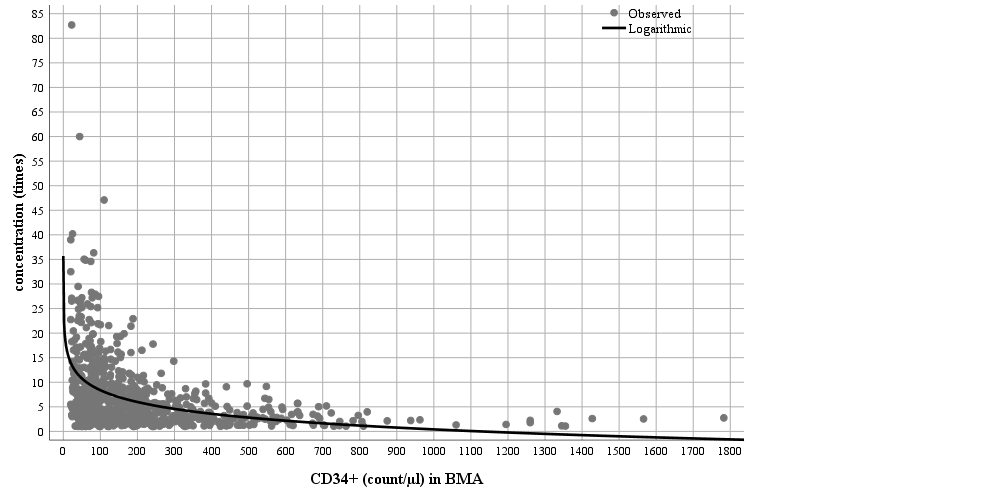
**

**FIGURE 4** CD34+ cells in bone marrow aspirate (BMA) and bone marrow aspirate concentrate (BMAC). Scatter plot showing that the lower the CD34+ cells (count/µl) in BMA the higher the concentration (times) of CD34+ cells (count/µl) in BMAC after centrifugation.

Since the onset of regenerative medicine several centrifuges are available on the market for mononuclear cell concentration.[4, 5] The concentration factor in our data showed a negative correlation to the number of leukocytes in BMA. Thus, a higher degree of concentration is found in patients with a lower bone marrow leukocyte count. This in part counteracts the dilution occurring in any aspirate higher than 2ml, which is due to the inflow of peripheral blood into bone marrow during the aspiration process.[6]

**REFERENCES**

1. Oliver, K., T. Awan, and M. Bayes, *Single- Versus Multiple-Site Harvesting Techniques for Bone Marrow Concentrate: Evaluation of Aspirate Quality and Pain.* Orthop J Sports Med, 2017. **5**(8): p. 2325967117724398.

2. Terstappen, L.W., et al., *Sequential generations of hematopoietic colonies derived from single nonlineage-committed CD34+CD38- progenitor cells.* Blood, 1991. **77**(6): p. 1218-27.

3. Brooimans, R.A., et al., *Flow cytometric differential of leukocyte populations in normal bone marrow: influence of peripheral blood contamination.* Cytometry B Clin Cytom, 2009. **76**(1): p. 18-26.

4. El-Jawhari, J.J., et al., *Enrichment and preserved functionality of multipotential stromal cells in bone marrow concentrate processed by vertical centrifugation.* Eur Cell Mater, 2020. **40**: p. 58-73.

5. Hegde, V., et al., *A prospective comparison of 3 approved systems for autologous bone marrow concentration demonstrated nonequivalency in progenitor cell number and concentration.* J Orthop Trauma, 2014. **28**(10): p. 591-8.

6. Hernigou, P., et al., *Benefits of small volume and small syringe for bone marrow aspirations of mesenchymal stem cells.* Int Orthop, 2013. **37**(11): p. 2279-87.
